# Making the Cut: A Six-Year "Bone-afide" Membership Trend From Student to Surgeon

**DOI:** 10.7759/cureus.76484

**Published:** 2024-12-27

**Authors:** Minali G Nemani, Emma Barham, Julieanne P Sees

**Affiliations:** 1 Biomedical Affairs and Research, Edward Via College of Osteopathic Medicine, Monroe, USA; 2 Haub School of Business, St. Joseph's University, Philadelphia, USA; 3 Orthopedic Surgery, American Osteopathic Association, Chicago, USA

**Keywords:** acgme, match, organization, orthopedics, osteopaths, residency

## Abstract

Background: Mentorship and early exposure vary greatly from school to school for osteopathic medical students. Historically, allopathic medical students have matched at a higher rate for competitive surgical specialties, like orthopedic surgery, compared to osteopathic medical students. The unique position of medical student interest organizations in filling those gaps and their related outcomes, including successful residency acceptance, is unknown.

Objective: The purpose of our study was to investigate whether membership in the Student Section of the American Osteopathic Academy of Orthopedics (SAOAO) plays a role in osteopathic medical student match success in orthopedic surgery residency programs.

Methods: This comprehensive observational study was conducted using six years of all public resident data collected from residency program websites and affiliated social media accounts of 200 Accreditation Council for Graduate Medical Education (ACGME)-accredited orthopedic surgery residency training programs and was cross-referenced to the National SAOAO membership directory for the 2019-2024 match cycles, as of August 2024. Data analysis of the 5112 data points was conducted using a two-proportion z-test with significance set at a p-value of less than 0.05. Dataset normality is assumed based on the Central Limit Theorem.

Results: In the 2024 orthopedic surgery match cycle, the data demonstrated 51.8% of matched osteopathic medical students were members of SAOAO, a statistically significant increase compared to previous years of 2019, 28.3%; 2020, 22.3%; 2021, 20.3%; 2022, 41.2%; and 2023, 19.4%. The change over 2019-2024 revealed a positive correlation with SAOAO membership in the orthopedic residency match results. There was a statistically significant increase in female osteopathic residents matching into orthopedic surgery between 2019 and 2024. Among the male osteopathic residents, the percentage of residents with prior SAOAO membership significantly increased from 22.1% in 2019 to 51.2% in 2024. SAOAO membership representation among female residents experienced a statistically significant decrease in 2020 and an increase in 2024. Also in 2024, 25.4% of matched SAOAO members were female. Broken down by gender, the male match rate was 56.3% and the female match rate was 60.0%. From 2018-2019 to 2023-2024, the SAOAO executive board experienced a statistically significant increase in representation of female board members of 12.5% (one of eight) to 62.5% (five of eight), respectively.

Conclusion: Our study trending six years of osteopathic orthopedic surgical match results from 200 ACGME residency programs indicates that over time SAOAO membership has had increased success with osteopathic medical students matching into orthopedic surgery residency programs. Looking at the increased match rates for those on the SAOAO executive board, leadership could be an important factor to consider in applicants. Additionally, SAOAO has been shown to provide an avenue for supporting and showcasing future female osteopathic orthopedic surgeons. Furthermore, continuing with this holistic approach of student engagement, education, and mentorship, future studies are planned to continue tracking trends including characteristics and activities that may provide valuable insight for career success with membership in surgical, medical, and professional academies and associations.

## Introduction

Specialty-specific interest organizations in healthcare have been avenues for aspiring professionals to gain early exposure, develop a network, and find mentorship to help better their chances of pursuit in that profession [1,2]. Awards, research publications [3], academic achievement, and recommendation letters all play a role in the match results for an applicant within the fields of medicine and surgery [4]. Since mentorship and early exposure [5] in surgical specialties, like orthopedic surgery, vary greatly between academic institutions for osteopathic medical students, it is crucial for them to remedy those differences from their allopathic counterparts to increase their chances of matching into that specific field of study.

In order for applicants to receive the prestigious invitation to interview and subsequent high-ranking by United States (US)-based residency programs, there are various subjective and objective criteria that program directors consider to narrow down the applicant pool [6]. For US osteopathically trained seniors applying to orthopedic surgery residency programs in 2022, having eight contiguous ranks of post-interview programs gave an applicant approximately an 80% chance of matching into that specialty [7]. Therefore, the benefits gained from specialty-specific interest organizations including granted awards, research publications, academic achievement, and recommendation letters to name a few are distinctions for which residency program applicants strive to have in order to showcase the optimal holistic candidate with ultimate match into their desired field of study [4].

Historically, allopathic medical students have matched at a higher rate for competitive surgical specialties, like general surgery, urology, and orthopedic surgery, compared to osteopathic medical students [8]. Specifically in orthopedic surgery, allopathic medical graduates have a match rate of 65.0% (4466 of 6869) and osteopathic medical graduates of 45.7% (737 of 1612) from 2019 to 2024 [9-11]. In this same time frame, 4,466 allopathic medical graduates and 737 osteopathic medical graduates were orthopedic surgery residents. Since osteopathic medical students have a more difficult time matching into orthopedic surgery, professional organizations like the American Osteopathic Academy of Orthopedics (AOAO) and related opportunities could be useful for osteopathic medical students seeking to match.

The AOAO established in 1941 with over 3,000 osteopathic physician (DO) members is recognized as the principal American academy for osteopathically board-certified orthopedic surgeons whose executive leadership continues steadfast commitment to supporting initiatives for ODs, residents, and medical students in order to thrive in orthopedic surgery and promote holistic professionalism throughout healthcare [12]. The AOAO serves an important mission to remedy the disparity in osteopathically trained physician representation in the field of orthopedics, while additionally building the future of our national healthcare workforce, critically impacting the care environment, employee retention, quality delivery, organizational sustainability, and valuable success with trust.

The unique position of student interest organizations, especially in medicine like the student section of AOAO (SAOAO), in filling the mentorship and networking gaps to create competitive applicants and the correlation with successful residency acceptance, is unknown. Interest organizations in the past have looked at general correlation with match results without a particular diversity initiative goal; however, studying the success of a professional healthcare organization such as the AOAO in supporting osteopathic medical students match into orthopedic surgery remains a profoundly interesting case to analyze [13,14]. The purpose of our study was to investigate whether membership in the academy-supported student section of the AOAO fulfills the academy’s mission of providing support to osteopathic medical graduates matching into orthopedics with an increased association of membership and matched US DO residents over a six-year time period of 2019-2024.

## Materials and methods

The local institutional review board deemed the study exempt from review because it was done using publicly available and privately identifiable data sources. This comprehensive observational study was conducted using six years of public resident data collected from residency program websites and affiliated social media accounts of 200 Accreditation Council for Graduate Medical Education (ACGME)-accredited orthopedic surgery residency training programs and was cross-referenced to the National SAOAO membership directory for the 2019-2024 match cycles, as of August 2024. While there were 209 accredited orthopedic residency programs in the 2023-2024 academic year, some programs, a majority being military residency programs, do not list their residents online and are therefore excluded from the study [15]. The resident's name, match year, medical school, and residency program were noted for comparison against the SAOAO membership directory. Incomplete resident data and residents from programs that did not publicly display resident information on residency websites and social media sites were excluded. This data was categorized by match cycle based on the assumption that the residents did not take time off or repeat a year of residency and that all the information online was up to date. Male and female designation was determined by observational determination and coding data into two categories. SAOAO membership was determined by having a join date prior to the calculated year they began residency (e.g., a match year 2024 orthopedic resident must have joined SAOAO/AOAO before January 2024).

Statistical analysis

The 5112 data points were collected and analyzed in MS Excel (Microsoft Corporation, Redmond, Washington, United States). Dataset normality is assumed due to the large sample based on the central limit theorem. Statistical analysis was computed using a two-proportion z-test using percentages and group sizes between gender groups and match years. Statistical significance is noted at a p-value of less than 0.05.

## Results

Increased SAOAO membership representation among residents

**Figure 1:**
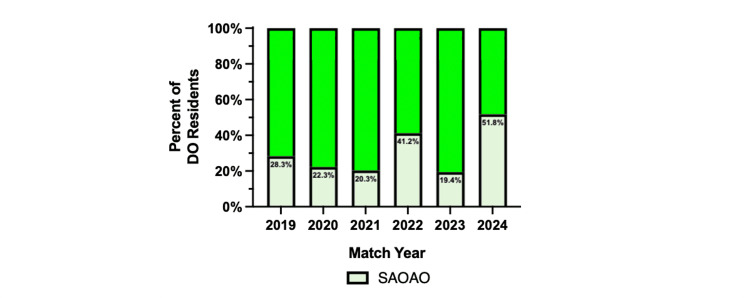
SAOAO membership of osteopathic orthopedic residents from 2019 to 2024 This data represents the percentage of all osteopathic (DO) residents with student section of the American Osteopathic Academy of Orthopedics (SAOAO) membership in each match cycle year representing their first postgraduate year in a five-year orthopedic surgery residency program accredited by the Accreditation Council for Graduate Medical Education (ACGME). For 2019, the total residents, N = 827; total DO residents, N = 120. For 2020, the total residents, N = 851; total DO residents, N = 130. For 2021, the total residents, N = 851; total DO residents, N = 118. For 2022, the total residents, N = 884; total DO residents, N = 119. For 2023, the total residents, N = 882; total DO residents, N = 124. For 2024, the total residents, N = 827; total DO residents, N = 114. Statistical analysis was computed using a two-proportion z-test where the statistical significance is noted at a p-value of less than 0.05

In 2024, the data demonstrates 51.8% of matched osteopathic medical students (59 of 114) were members of SAOAO, a statistically significant (p < 0.05) increase compared to previous years: 2019, 28.3% (34 of 120); 2020, 22.3% (29 of 130); 2021, 20.3% (24 of 118); 2022, 41.2% (49 of 119); and 2023, 19.4% (24 of 124) (Figure 1). SAOAO membership representation among DO orthopedic surgery residents experienced a statistically significant (p < 0.05) increase in 2022, a decrease in 2023, and an increase in 2024. Osteopathic medical graduates make up on average 120 orthopedic residents each match year, while matched SAOAO members make up on average 36.5 orthopedic residents. The years 2021 and 2023 had the lowest number of SAOAO members being 24 residents matching into orthopedic surgery. The year 2024 has the highest being 59 residents matching with SAOAO membership. DO orthopedic residents with SAOAO membership more than doubled from 2023 to 2024.

Gender distribution of DO residents

**Figure 2:**
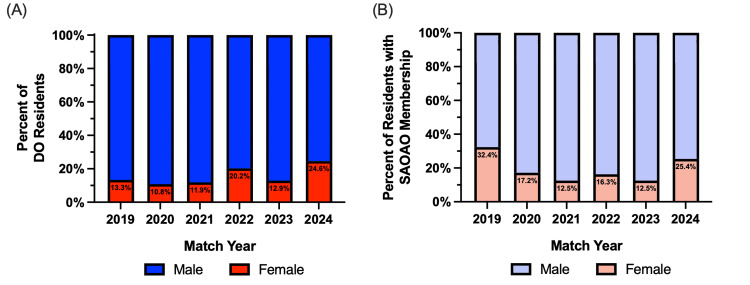
The 2019-2024 gender distribution among osteopathic orthopedic residents (A) and those residents with SAOAO membership (B) This data in A represents the percentage of all osteopathic (DO) residents who are female in each match cycle year representing their first postgraduate year in a five-year orthopedic surgery residency program accredited by the Accreditation Council for Graduate Medical Education (ACGME). For 2019, the total residents, N = 827; total DO residents N = 120. For 2020, the total residents, N = 851; total DO residents, N = 130. For 2021, the total residents, N = 851; total DO residents, N = 118. For 2022, the total residents, N = 884; total DO residents, N = 119. For 2023, the total residents, N = 882; total DO residents, N = 124. For 2024, the total residents, N = 827; total DO residents, N = 114. This data in B represents the percentage of osteopathic (DO) residents with the student section of the American Osteopathic Academy of Orthopedics (SAOAO) membership who are female residents in each match cycle year representing their first postgraduate year in a five-year orthopedic surgery residency program accredited by the Accreditation Council for Graduate Medical Education (ACGME). For 2019, the total residents N = 827, total residents with SAOAO membership N = 34. For 2020, the total residents N = 851, total residents with SAOAO membership N = 29. For 2021, the total residents N = 851, total residents with SAOAO membership N = 24. For 2022, the total residents N = 884, total residents with SAOAO membership N = 49. For 2023, the total residents N = 882, total residents with SAOAO membership N = 24. For 2024, the total residents N = 827, total residents with SAOAO membership N = 59. Statistical analysis was computed using a two-proportion z-test where statistical significance is noted at a p-value of less than 0.05

From the data collected, of the 120 DO residents in 2019, 130 DO residents in 2020, 118 in 2021, 119 in 2022, 124 in 2023, and 114 in 2024, female DO residents made up 13.3% of the 2019 orthopedic surgery residents, 10.8% in 2020, 11.9% in 2021, 20.2% in 2022, 12.9% in 2023, and 24.6% in the 2024 residents. There was a statistically significant (p < 0.05) increase in female DO residents matching into orthopedic surgery between 2019 and 2024 (Figure 2A). Like the increase seen in SAOAO membership among DO residents in 2022 (Figure 1), there was a statistically significant (p < 0.05) increase in female DO residents. While the decrease in 2023 among female DO residents was not statistically significant, the increase in 2024 was. The match year with the highest percentage of female DO residents was 2024. The match year with the lowest percentage of female DO residents was 2020. The gender distribution of female DO orthopedic residents with SAOAO membership is 32.4% in the 2019 residents with SAOAO membership, 17.2% in 2020, 12.5% in 2021, 16.3% in 2022, 12.5% in 2023, and 25.4% in the 2024 residents with SAOAO membership. When comparing the gender distribution of DO orthopedic residents to those with SAOAO membership, 2019 had a statistically significant (p < 0.05) increase in female representation with 32.4% of matched SAOAO members (Figure 2b). There were no statistically significant differences in gender distribution between 2019 and 2024 for SAOAO member residents. The match year with the highest percentage of female residents among those with SAOAO membership was 2019 with 32.5%. The match years with the lowest percentage of female residents among those with SAOAO membership were 2021 and 2023 with 12.5%.

Male and female residents' distribution with SAOAO membership

**Figure 3:**
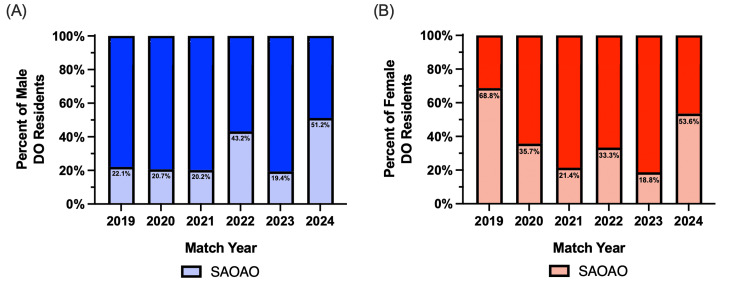
The 2019-2024 male (3A) and female (3B) distribution of osteopathic orthopedic residents matching with SAOAO membership A represents the percentage of all male osteopathic (DO) residents who have student section of the American Osteopathic Academy of Orthopedics (SAOAO) membership in each match cycle year representing their first postgraduate year in a five-year orthopedic surgery residency program accredited by the Accreditation Council for Graduate Medical Education (ACGME). For 2019, the total residents, N = 827; total male DO residents, N = 104. For 2020, the total residents, N = 851; total male DO residents, N = 116. For 2021, the total residents, N = 851; total male DO residents, N = 104. For 2022, the total residents, N = 884; total male DO residents, N = 95. For 2023, the total residents, N = 882; total male DO residents, N = 108. For 2024, the total residents, N = 827; total male DO residents, N = 86. This data in B represents the percentage of all female osteopathic (DO) residents who have the student section of the American Osteopathic Academy of Orthopedics (SAOAO) membership in each match cycle year representing their first postgraduate year in a five-year orthopedic surgery residency program accredited by the Accreditation Council for Graduate Medical Education (ACGME). For 2019, the total residents, N = 827; total female DO residents, N = 16. For 2020, the total residents, N = 851; total female DO residents, N = 14. For 2021, the total residents, N = 851; total female DO residents, N = 14. For 2022, the total residents, N = 884; total female DO residents, N = 24. For 2023, the total residents, N = 882; total female DO residents, N = 16. For 2024, the total residents, N = 827; total female DO residents, N = 28. Statistical analysis was computed using a two-proportion z-test where statistical significance is noted at a p-value of less than 0.05

The distribution of male DO orthopedic residents with SAOAO membership is 22.1% in the 2019 male DO residents (23 of 104), 20.7% in 2020 (24 of 116), 20.2% in 2021(21 of 104), 43.2% in 2022(41 of 95), 19.4% in 2023 (21 of 108), and 51.2% in the 2024 (44 of 86) male DO residents. Among the male DO orthopedic residents, the percentage of residents with prior SAOAO membership had a statistically significant (p < 0.05) increase from 22.1% in 2019 to 51.2% in 2024 (Figure 3a). SAOAO membership representation among male DO orthopedic surgery residents experienced a statistically significant (p < 0.05) increase in 2022, a decrease in 2023, and an increase in 2024. This trend of statistical significance from 2022 to 2024 was also seen in SAOAO membership in Figure 1. The match year with the highest percentage of male DO residents having SAOAO membership was 2024 with 51.2%. The match year with the lowest percentage of male DO residents having SAOAO membership was 2023 with 19.4%.

The distribution of female DO orthopedic residents with SAOAO membership is 68.8% in the 2019 (11 of 16) female DO residents, 35.7% in 2020 (five of 14), 21.4% in 2021 (three of 14), 33.3% in 2022 (eight of 24), 18.8% in 2023 (three of 16), and 53.6% in the 2024 (15 of 28) female DO residents. From the data collected, there was no statistically significant change in the percentage of female DO residents with SAOAO membership matching with orthopedic surgery between 2019 and 2024 (Figure 3b). SAOAO membership representation among female DO orthopedic surgery residents experienced a statistically significant (p < 0.05) decrease in 2020 and an increase in 2024. The match year with the highest percentage of female DO residents having SAOAO membership was 2019 with 68.8%. The match year with the lowest percentage of female DO residents having SAOAO membership was 2023 with 18.8%.

SAOAO leadership matches at a higher rate

**Figure 4:**
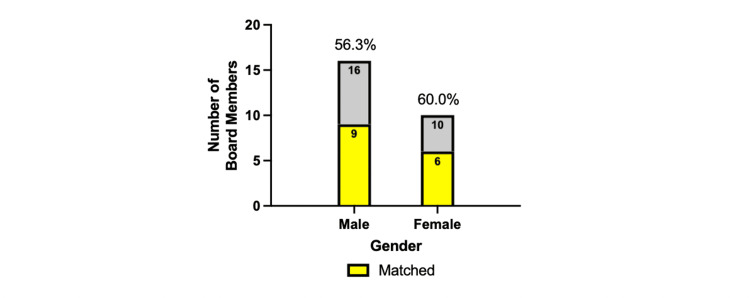
Orthopedic match by gender among SAOAO executive board 2019-2024 This data represents the percentage of the unique student section of the American Osteopathic Academy of Orthopedics (SAOAO) executive board members matching in a five-year orthopedic surgery residency program accredited by the Accreditation Council for Graduate Medical Education (ACGME) separated by male and female. All residents collected were N = 5112, N = 25 were male executive board members, and N = 16 were female executive board members for SAOAO who have applied for the match process in the 2019-2024 match cycles. Statistical analysis was computed using a two-proportion z-test where statistical significance is noted at a p-value of less than 0.05

With a total of 26 unique SAOAO executive board members from 2019 to 2024, 16 were male and 10 were female (Figure 4). The overall match rate for SAOAO executive board members was 57.69%. Broken down by gender, the male match rate was 56.3%, and the female match rate was 60.0%. From 2018-2019 to 2023-2024, the SAOAO executive board experienced a statistically significant increase in representation of female board members of 12.5% (one of eight) to 62.5% (five of eight), respectively.

## Discussion

It has become increasingly difficult for osteopathic medical students to match into orthopedic surgery despite increased interest [9]. Osteopathic medical students have been identified to pursue primary care specialties at higher rates as a reason for the lower osteopathic medical graduate match rates into surgical specialties [8]; however, each year there is an increase in osteopathic orthopedic surgery residency applicants without a subsequent increase in DO orthopedic match rates [8-10]. This phenomenon is a hot topic of research currently with not many direct answers provided as to the particular cause as to why osteopathic medical graduates match at a lower rate. Therefore, it is imperative that osteopathic medical graduates seek mentorship and guidance through organizations closely involved with residency programs that predominantly take osteopathic medical students like the AOAO. Thus, observing trends in DO match rates with respect to SAOAO membership is of great interest to help establish evidence that SAOAO membership directly impacts match rates. Specifically, match rates of female applicants should be closely observed to better understand the nature of the impact membership has on match rates. The increasing number of osteopathic orthopedic residents holding an SAOAO membership as a medical student demonstrates a positive relationship with match outcomes and may be an avenue to help DO medical students remedy a part of that gap in a match. Among our SAOAO members who go on to become matched orthopedic residents, there is a higher representation of females, and this may prove to be a critical pipeline for female DO medical students to participate in. Female residents represent approximately 20% of all orthopedic surgery residents, and the female DO residents were 1.8% in 2019-2023 [16]. Having an intersectional identity of being an osteopath and a female as an applicant in orthopedic surgery makes it increasingly difficult to find an avenue for advising and mentorship that can be considerate of both identities and their associated barriers to match.

The objective measurements of applicant success in awards, research publications, academic achievement, and recommendation letters have been researched and proven to be valuable for applicant success; however, leadership roles in a specialty interest group like SAOAO and any correlation to match rates in orthopedics have not been clearly reported in literature. From the six years of SAOAO executive boards, our study demonstrates a higher match rate among executive board members compared to general DO applicants. The higher female DO distribution among residents with SAOAO membership and SAOAO executive board members leads to the conclusion that the AOAO concurrently supports women matching into orthopedic surgery, through various initiatives like events put on by the Women’s Section of AOAO [17]. Therefore, the direct and indirect benefits brought forth by leadership roles and networks within AOAO may be a valuable indicator of a DO applicant’s potential success in matching into orthopedics, especially for females. Given that SAOAO membership alone could potentially lead to greater outcomes in the match process for DO students, leadership roles in SAOAO and their correlation to match rates are of interest as well. Leadership roles allow DO students pursuing orthopedics an upper hand on various aspects of the match process, such as providing more opportunities to get involved with research publications, gain access to more personal letters of recommendation, etc. This notion is further supported by the higher-than-average match rate for both male and female DO SAOAO executive board members.

The matching process is ever-changing for medical students with such events in the past six years to include an international COVID-19 pandemic, a single accreditation system implemented in 2020, and the introduction of signaling in 2023. The COVID-19 pandemic led to a temporary shift in program director interest in virtual opportunities and social media when selecting orthopedic surgery residents for their programs [18]. Regarding SAS, it expanded opportunities for DO students while simultaneously intensifying competition for many specialties such as orthopedics [16]. The introduction of signaling has led to a narrowed focus of applications sent out to focus on effective application cycles [19]. It is important now more than ever for medical students to seek mentorship and guidance through the orthopedic surgery residency application process through dedicated organizations and develop networks to guide them through each recent update in the process and the associated impact on match trends.

For the osteopathic medical profession, the key is in the philosophy which consists of four tenets creating the foundation for osteopathic medicine’s whole-person approach to health while simultaneously influencing each individual, professional, and organizational character within the healthcare atmosphere. The osteopathic tenets include the following: (1) The body is a unit with the person being a unit of body, mind, and spirit. (2) The body is capable of self-regulation, self-healing, and health maintenance. (3) Structure and function are reciprocally interrelated. (4) Rational treatment is based upon an understanding of the basic principles of body unity, self-regulation, and the interrelationship of structure and function [20]. Patient-centered approach to health leverages these tenets where Doctors of Osteopathic Medicine/DOs are specifically trained to engage with and listen to their patients looking beyond symptoms to lifestyle choices that may impact a patient's health or recovery. With such an advantage, the osteopathic approach to care then resonates within its membership, such as in our study from students to surgeons as in the SAOAO to AOAO, and ultimately leadership leverages to conduct business and promote successful governance across professional societies and the entire healthcare ecosystem. Our data support the traditional aim of the AOAO at more comprehensive inclusivity with the promotion of academic training nationally in addition to home institution and leader mentorship to enhance quality within the professional organization and the essential workforce for the diverse communities in which they traditionally serve.

Limitations

There are limitations to any study. Specifically, in terms of the methodology, one limitation to understand in this study is that there are resident data points that have not been published online, as this could change our percentages for each match cycle under each category. Similarly, the data published online is assumed to be correct and up to date by the program. Another limitation is that the analysis and retroactive categorization of residents by match cycle operated under the assumption that residents do not take extra time during residency; however, residents may take time off because of a multitude of personal reasons like pregnancy, research, etc. Finally, a limitation is that we are unable to account for name changes when identifying membership within the AOAO directory, since the student membership may not be updated as residents tread through residency. With these limitations under consideration, the study has numerous reliable data points to rely on for a robust research data set to analyze for statistical significance. 

When developing conclusions based on our collected data, it is important to consider the many confounding factors that can result in an applicant's match outcomes into an ACGME-accredited orthopedic surgery residency program. Individuals who may seek out involvement in specialty-specific healthcare organizations may possess certain characteristics to seek out numerous activities that align with various factors considered in residency applicants like recommendation letters and research opportunities [4]. Membership in SAOAO/AOAO should be not considered a direct indicator of a match into orthopedic surgery. However, increased female representation among those who matched with organization membership and increased membership prevalence among osteopathic orthopedic residents demonstrate that specialty-specific organizations can be seen as an avenue to provide the competitive factors coveted by residency programs, especially in those demographic groups that have shown to have less representation in the field.

## Conclusions

Our unique study trending six years of osteopathic orthopedic surgical match results from 200 residency programs indicates that over time SAOAO membership has had an increased success with osteopathic medical students matching into orthopedic surgery residency programs. Additionally, SAOAO has been shown to provide an avenue for supporting and showcasing future female osteopathic orthopedic surgeons. Furthermore, continuing with this holistic approach of student engagement, education, and mentorship, future studies are planned to continue tracking trends including characteristics and activities that may provide valuable insight for career success with membership in surgical, medical, and professional academies and associations. To enhance quality healthcare, it is crucial to take action considering this model toward inclusivity in training and mentorship, promoting diversity not only within orthopedics but the entirety of the medical landscape in ensuring a balanced, healthy ecosystem serving the diverse patient populations within our communities and promotion of the fruitful success of a professional healthcare organization.
